# On the Molecular Evolution of Leptin, Leptin Receptor, and Endospanin

**DOI:** 10.3389/fendo.2017.00058

**Published:** 2017-04-10

**Authors:** Richard Lyle Londraville, Jeremy W. Prokop, Robert Joel Duff, Qin Liu, Matthew Tuttle

**Affiliations:** ^1^Program in Integrative Bioscience, Department of Biology, University of Akron, Akron, OH, USA; ^2^Hudson Alpha Institute for Biotechnology, Huntsville, AL, USA

**Keywords:** leptin, leptin receptor, endospanin, *in silico* modeling, molecular evolution, obesity, adipostat, fish models

## Abstract

Over a decade passed between Friedman’s discovery of the mammalian leptin gene (1) and its cloning in fish (2) and amphibians (3). Since 2005, the concept of gene synteny conservation (vs. gene sequence homology) was instrumental in identifying leptin genes in dozens of species, and we now have leptin genes from all major classes of vertebrates. This database of *LEP* (leptin), *LEPR* (leptin receptor), and *LEPROT* (endospanin) genes has allowed protein structure modeling, stoichiometry predictions, and even functional predictions of leptin function for most vertebrate classes. Here, we apply functional genomics to model hundreds of LEP, LEPR, and LEPROT proteins from both vertebrates and invertebrates. We identify conserved structural motifs in each of the three leptin signaling proteins and demonstrate *Drosophila* Dome protein’s conservation with vertebrate leptin receptors. We model endospanin structure for the first time and identify endospanin paralogs in invertebrate genomes. Finally, we argue that leptin is not an adipostat in fishes and discuss emerging knockout models in fishes.

## Introduction

In 1994, Friedman’s laboratory described leptin as a peptide hormone that is synthesized by adipose tissue (1) and soon after it was proposed to regulate appetite and metabolic rate by communicating energy stores to the central nervous system (4–6). In mammals, leptin is synthesized by adipose tissue and released into the blood; there it travels to the hypothalamus and binds to the leptin receptor, which stimulates reduction of appetite and increased mobilization of lipid for metabolism. Through this feedback loop, the brain regulates energy stores to remain relatively constant [“adipostat control” (4–6)]. Control of energy stores is central to an organism’s life history, and as such, it is a research focus for comparative biologists. Migratory birds fuel long-distance migration by dramatic changes in lipid stores (7), hibernating mammals accumulate lipid stores to survive long periods of torpor (8), snakes dramatically rework organs to process large and infrequent meals (9), amphibian survival after metamorphosis is tied to adipose stores (10), and fish routinely go months without eating during winter (11). Agricultural scientists also have a great interest in leptin, because manipulating energy acquisition and deposition has potential to influence production of commercially important species (12–14). Therefore, there has been great interest and effort expended toward cloning and characterizing leptin orthologs throughout vertebrates. Recent reviews thoroughly document the progress of the comparative leptin community (15–17). This review will focus on three areas: evolution of genes in the leptin signaling pathway, the status of leptin as an adipostat, and emerging non-mammal models for studying leptin signaling. These research topics have made significant progress in recent years, and they provide examples of how a comparative approach can inform the study of human leptin (hLEP) endocrinology.

## Evolution of Leptin Signaling: Leptin and Leptin Receptor Genes among Vertebrates

**Figure 1:**
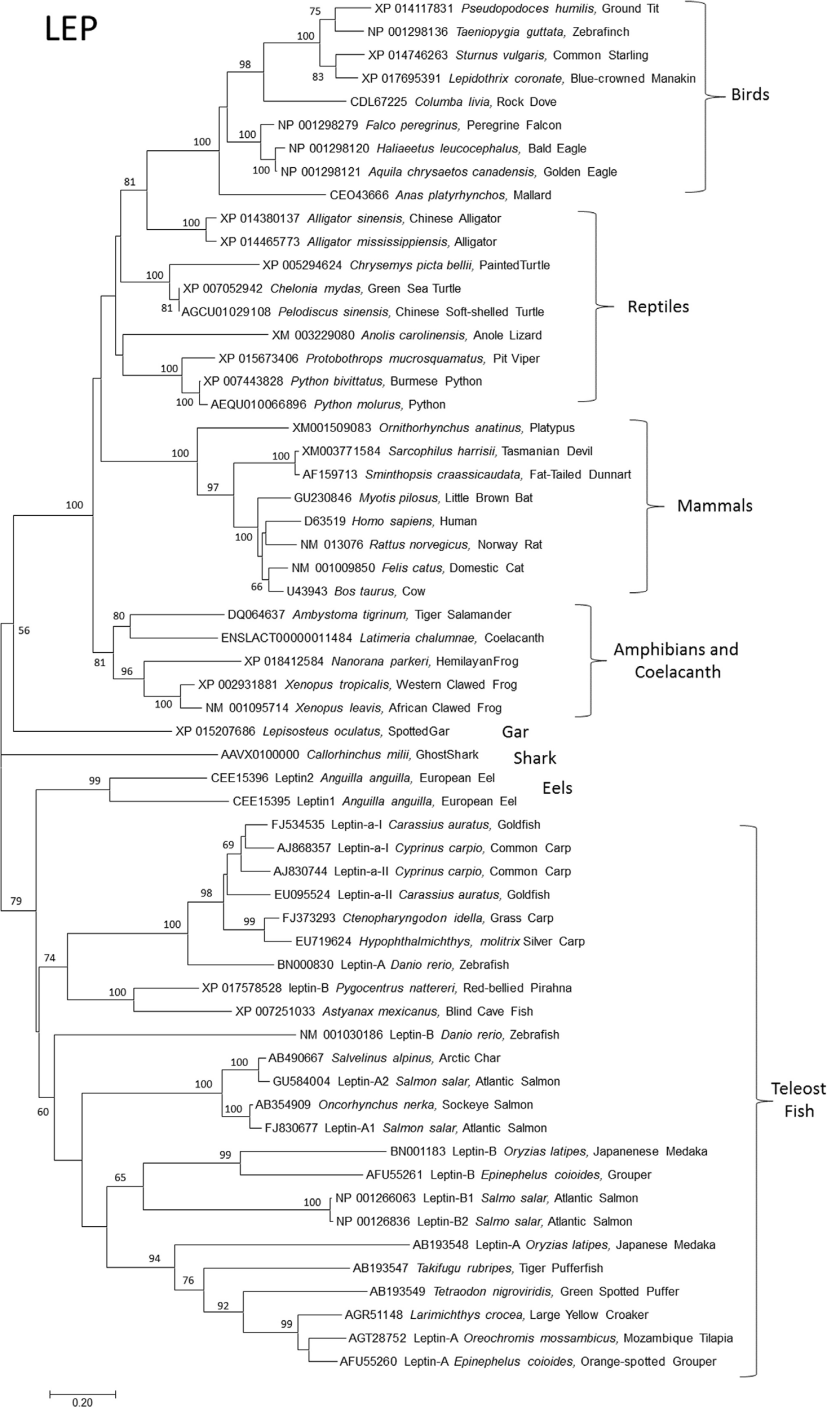
**Evolutionary relationships of vertebrate leptins (LEPs)**. Relationships of 59 amino acid sequences using the neighbor-joining method conducted in MEGA7. Numbers at nodes represent percentage of 500 bootstrap replicates. Nodes with no number indicate bootstrap support of less than 50%. Leptin amino acid sequences were manually aligned in MEGA7 informed by protein structural homologies. GenBank accession numbers represent protein accession.

**Figure 2:**
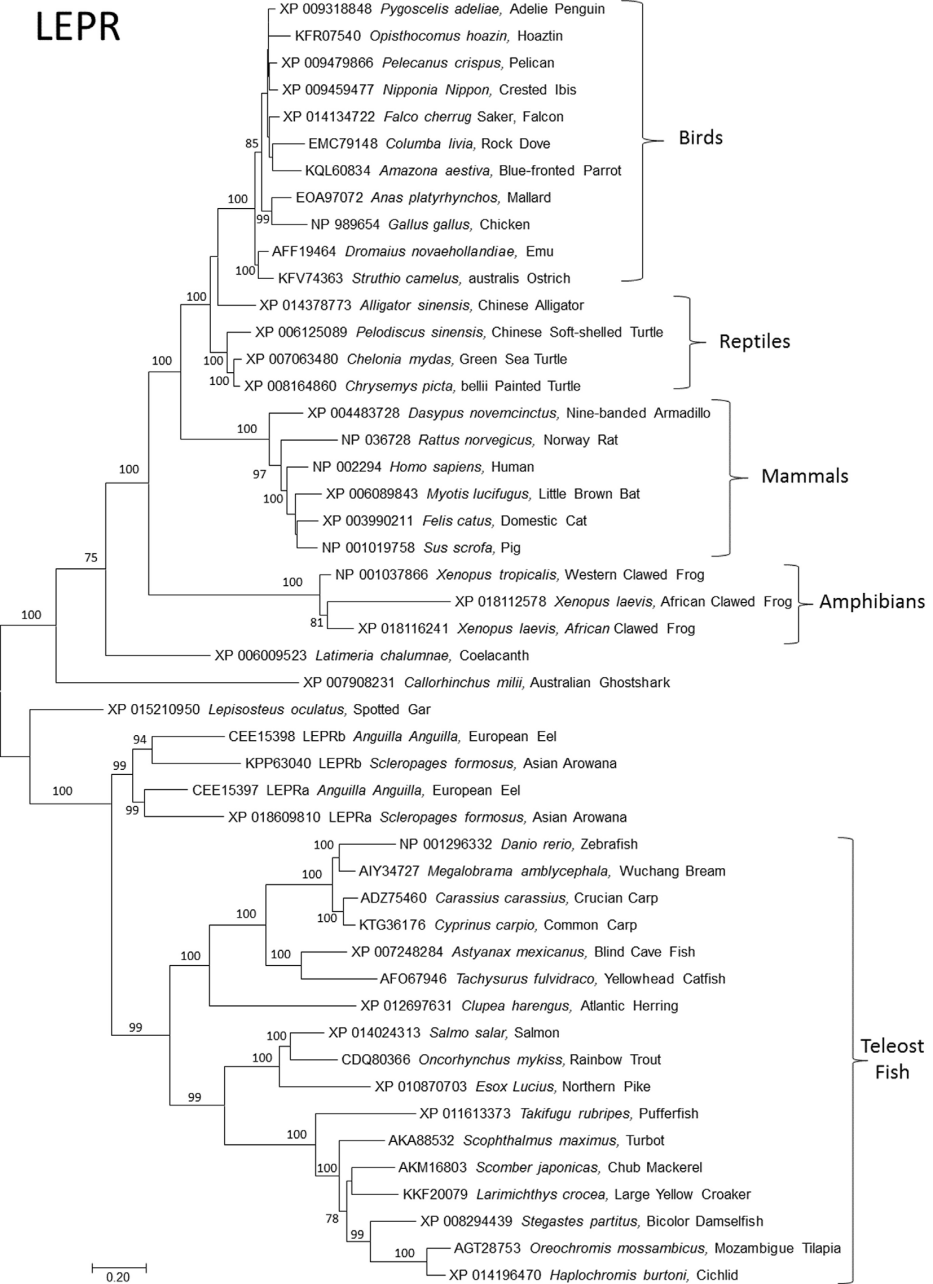
**Evolutionary relationships of vertebrate leptin receptor (LEPR)**. Relationships of 48 amino acid sequences using the neighbor-joining method conducted in MEGA7. Numbers at nodes represent percentage of 500 bootstrap replicates. Nodes with no number indicate bootstrap support of less than 50%. GenBank accession numbers represent protein accessions.

Although leptins in domestic mammals were identified soon after leptin in mice (18, 19), the first non-mammal leptin gene took over a decade to discover (2). This was due to the false assumption of sequence conservation among orthologs and was overcome by Kurokawa’s insight of gene order conservation or synteny (2). This major advance, along with progress on genome projects, has allowed identification of *LEP*s and *LEPR*s in all classes of vertebrates (Figures 1 and 2). It is now clear that the ancestral leptin that gave rise to leptins in tetrapods (birds, reptiles, amphibians, and mammals) is more closely related to coelacanth and shark (*Callorhinchus milii*) leptins vs. leptins from bony fish. In other words, bony fish leptins diverged along their own lineage independent of leptins in higher mammals (Figures 1 and 2). After the bony fish and tetrapods diverged, multiple paralogs of fish *lep* evolved. Tetrapods and their closest living relatives for which we have data (gar, coelacanth, Dipnoi not determined) express a single ortholog of leptin (Figure 1), with the exception of *Anolis* lizard, which has two *lep*, one of which is not expressed (15).

Bony fish typically expresses two paralogs of leptin, referred to as “A” and “B.” These are interpreted as arising during the whole genome duplication event in the Teleost fish lineage; more recent duplications (in salmonids and carps) are subtypes of A and B [see the study by Morini et al. (20) for an insightful discussion of leptin paralog evolution]. Leptin receptors are typically present as single orthologs per species, with the exception of recently identified duplicate *lepr* paralogs in European eel (20) and Asian arowana (*Scleropages formosus*) (acc# XP 018609810 and KPP63040). This duplication event appears to be ancient, but it is unresolved if the duplication of *lepr* was present in the ancestor of teleost fishes and then lost, or if loss of *lepr* predates teleosts (Figure 2).

Amphibians express a single paralog of *lep* and *lepr*, with *lep* expressed in multiple tissues, including adipose (3, 21). *Xenopus* responds to homologous recombinant leptin as an anorexigen, but not at all life stages (3). *Xenopus* leptin stimulates hind limb (3) and lung (22) development and may influence life history decisions in spadefoot toad (23). *Xenopus lepr* binds homologous and non-homologous leptins (3) and stimulates phosphorylation of intracellular signal transducer and activator of transcription (STAT) 3 and 5 (24). Less is known about reptile leptins. Several reports indicate that reptiles respond to non-homologous leptin treatment consistent with the mammalian model of leptin function [e.g., reduced appetite (25), reproductive effects (25, 26)]. In addition, studies using non-homologous leptin antibodies have documented leptin-like proteins that respond to seasonal changes in lipid (27–29), which are consistent with mammalian models. Denver et al. reported 2 *lep* (one which may be non-functional) and 1 *lepr* in the genome of the green anole (15). In general, amphibian *lep* and *lepr* expression and *in vitro* and *in vivo* function are more consistent with mammalian models than are similar data for fishes and birds.

What is the significance of multiple leptin paralogs? We assert that leptin-A and -B paralogs have distinct functions in teleosts. The fact that both paralogs (in multiple species of teleosts) are maintained throughout the teleost lineage (Figure 1) argues that each paralog has a distinct function. Where expression has been measured, A-type *lep*s are typically expressed at higher message copies and with a more narrow tissue distribution than B-type (16, 30–33), but not in all species (2). If leptin-B paralogs are functional (and not pseudogenes), why is their expression lower and less tissue specific than A? Perhaps leptin-Bs are acting in an autocrine/paracrine manner, similar to that proposed for bird leptin (see below). Supporting this hypothesis is the observation that leptin-B is dramatically upregulated during regeneration of fin and heart in zebrafish (34), and after retinal injury (35), perhaps indicating local vs. circulating action. In addition, leptin-A knockdown in zebrafish embryos (*via* morpholino oligonucleotide) does not elicit a change in expression of leptin-B (36). If the A and B paralogs overlap functionally, one would expect a compensatory increase in B with decreased expression of A. Finally, *in silico* binding simulation of both paralogs predicts significantly lower binding energy of B vs. A to the leptin receptor (37). This indicates that something about the ligand–receptor interaction is different for leptin-B; perhaps it requires a second ligand or a higher local concentration of ligand (as in autocrine/paracrine signaling). To our knowledge, there are no published data on leptin-B protein expression or *in vivo* function other than regeneration (34–35). A leptin receptor reporter assay to assess functional differences between leptin paralogs, similar to that developed for *Xenopus* (24), and specific antibodies to document expression would be useful in addressing these questions.

## Evolution of Leptin Signaling: Is There Another Major Signaling System for Energy Stores in Birds?

Arguably, bird leptin was the most difficult to identify among vertebrates, with over a decade of significant effort from multiple laboratories. A purported chicken leptin gene was reported early on, but independent laboratories were unable to amplify the sequence from chicken tissues, and it was absent in early builds of the chicken genome, despite the presence of a leptin receptor (38–40). The missing bird leptin gene was eventually found within regions of genomes that were refractory to characterization due to their high GC content and repetitive sequence (41–44). The advent of new methods of whole genome sequencing allowed identification of bird leptin in most major lineages of birds. Recently (45), RNAseq experiments in chicken documented highest leptin transcript copy number in brain (hypothalamus and cerebrum) and pituitary, with moderate expression in pancreas and testis, and low expression in liver and adipose [typically high expressing leptin tissues in fish and mammals, respectively (16)]. Further, Friedman-Einat’s group speculated that the high correlation between leptin and leptin receptor transcripts indicated that leptin in birds may not circulate, but instead acts as an autocrine/paracrine factor (45). Several lines of evidence support this hypothesis: bird leptin expression is primarily in CNS (42, 45), bird blood did not activate a sensitive chicken leptin receptor assay, even in birds with extreme adiposity (46), and genes with high GC content (such as bird leptin genes) are associated with low transcription rates (47). One study that supported a circulating leptin in birds documented that chicken serum and crow blood caused translocation of GFP-labeled STAT 3 to the nucleus in an expressed chicken leptin receptor assay (48). However, potent leptin receptor antagonists tested in chickens effectively block chicken leptin receptor *in vitro* but not *in vivo* (49).

The primary sequences of bird leptins have typically low but recognizable homology with other vertebrate leptins (41, 45), and bird leptin primary structure folds *in silico* into the conserved tertiary structure seen in all leptins (44, 45). Despite this structural conservation (or homology), it otherwise appears that bird leptins do not function similar to leptins in other vertebrates (detailed above). We assert that leptin signaling in birds is fundamentally different than it is in other vertebrates, which suggests that there is a leptin-independent pathway to manipulate energy stores. Birds make large-magnitude changes in adipose stores routinely as a life history strategy. Red knots undergo massive changes in body composition during their 9,000-km migration flights (7), emperor penguin lose ~50% of their body mass during a 4-month fast while incubating eggs on ice (50), and ptarmigan accumulate up to 30% of body mass as lipid in anticipation of winter storms (51). If leptin signaling is reduced/altered in birds, what signals these dramatic changes in lipid mobilization? Other major mammalian adipokine/appetite genes are missing in chickens, including resistin, TNFα, serpine 1, and omentin (52), and ghrelin in falcons (53). Thus, the “usual suspects” for neuroendocrine control of energy stores are either absent or play a fundamentally different role (52).

## Evolution of Leptin Signaling: Analysis of Tertiary Structures Determined *In Silico*

**Figure 3:**
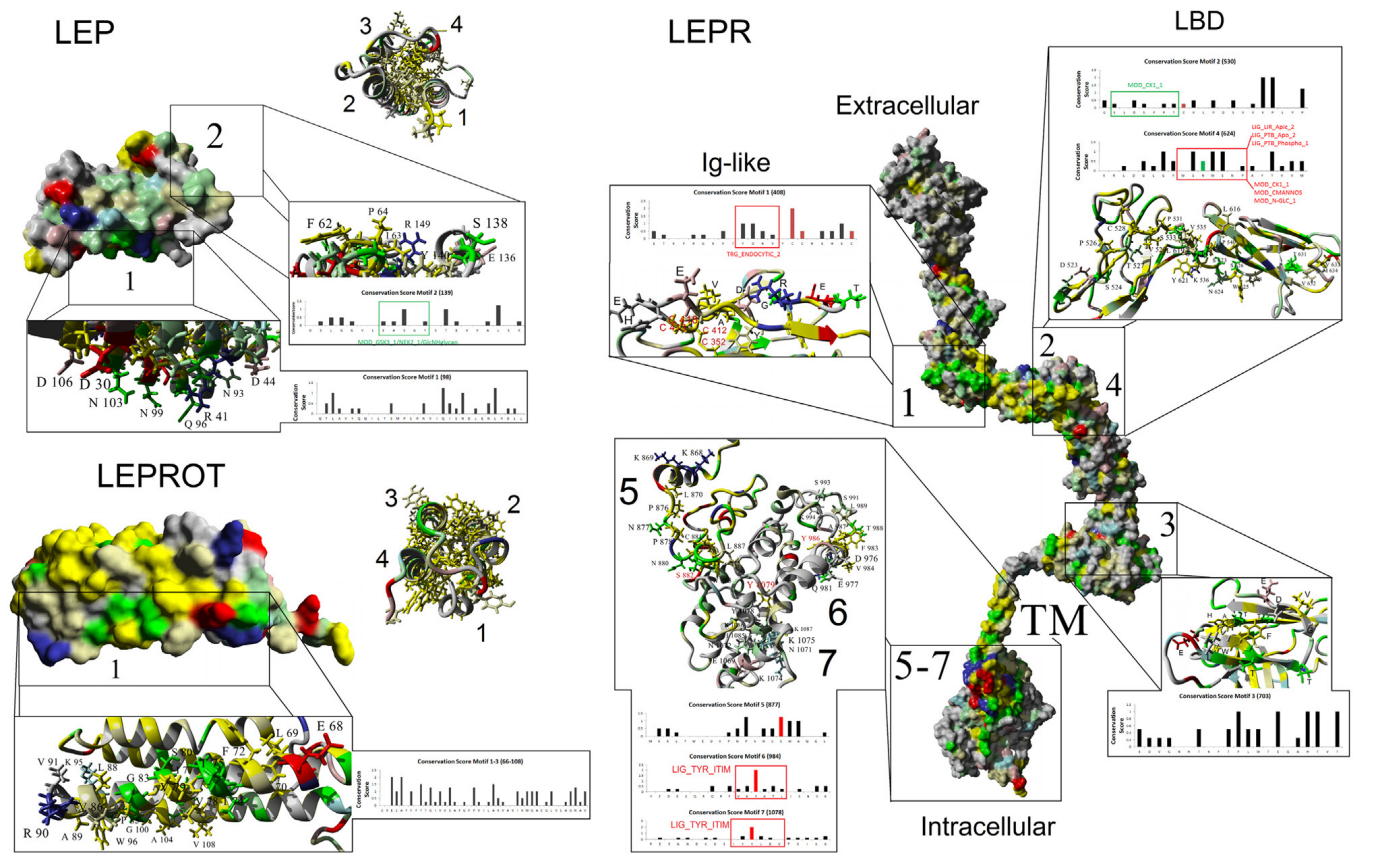
**Mapping protein conservation of leptin (LEP), leptin receptor (LEPR), and LEPROT/endospanin**. Consurf analysis of LEP (top left), LEPR (right), and LEPROT/endospanin (bottom left) are shown as molecular surface plots of each structure. For LEP and LEPR, a picture of the four-helix bundle with conserved hydrophobic amino acids is shown as a ribbon diagram beside the surface plots of conservation. Top conserved motifs are magnified, identifying conserved amino acids that contribute to each motif. Amino acids are colored as followed: yellow, conserved hydrophobic; red, conserved polar acidic; blue, conserved polar basic; green, conserved hydrophilic; gray, not conserved. Amino acids with known posttranslational modifications are red (disulfide bonds of Cys-C or phosphorylation of Ser-S/Thr-T/Tyr-Y) and green (glycosylation of Asn-N) on the bar graphs of conservation. Predicted eukaryotic linear motifs are boxed and labeled on the bar graphs.

In the effort to understand the evolution of vertebrate leptin function, often the first data available are sequence data, and we have used these data to model leptins, leptin receptors, and their interaction. Comparing ~100 primary sequences per gene (Table 1), we can make some generalizations about structure. Vertebrate leptins demonstrate considerable primary amino acid sequence divergence, but despite this retain high tertiary structure conservation (predicted) when modeled with the hLEP structure (15, 37). We analyzed multiple tertiary structures (generated *via in silico* modeling) and proposed conservation of critical binding sites between leptin and the leptin receptor from fish to human (37). Combining our previous work (37) with our sequence-to-structure-to-function tools (63), we addressed the vertebrate evolution of *LEP, LEPR*, and the lesser-studied *LEPROT*. By using a total of 93 vertebrate *LEP* sequences and 89 vertebrate *LEPR* sequences (Table 1), we mapped conservation and linear motifs for each gene onto protein structures (Figure 3). Leptins contain a conserved disulfide bridge (Table 1) and several hydrophobic amino acids that are critical to maintaining the four-helix packing of the protein, even though sequence homology is low (~20%). On the surface of leptins, two linear motifs were identified, one for interaction with the Ig-like domain as suggested by Peelman et al. (64) and the other for the leptin-binding domain of *LEPR*. Utilizing molecular modeling and dynamics, we studied the structural integrity of the leptin protein among many taxa and determined that while sequence is highly divergent, the conservation of several hydrophobic amino acids and the disulfide bridge is sufficient to maintain protein folding in all classes of vertebrates. The leptin receptor conserves protein folding with seven highly conserved and selected linear motifs. There are also 16 conserved sites for posttranslational modification within the receptor (Table 1).

*Concise Methods*: Open reading frame (ORF) sequences were obtained for each gene from NCBI gene and aligned to the human ORF using ClustalW (54) in Mega (55). Codon selection was calculated using HyPhy (56) under a Muse-Gaut model (57) and standard Tamura-Nei model (58) for all sites in the *LEP, LEPR, LEPROT*, and *LEPROTL1*. Conservation scores were calculated using a combination of codon/amino acid fixation rates and dN-dS scores of selective pressure. A score of 2 at any site implies both a greater than 2 SDs above the mean for codon selection and a site that an amino acid is 100% conserved. A score of 0 implies no conservation of the amino acid and below the mean selective pressure (dN-dS). The scores were then put on a sliding window of 21 codons to calculate the top linear motifs within each gene. All numbering throughout the article is based on the full gene sequence of human.

Protein modeling was performed using our previously published LEP–LEPR interaction model (37) combined with I-TASSER- (59) generated extracellular and intracellular domains of LEPR joined by a single-pass transmembrane helix. The endospanin proteins were modeled with I-TASSER (59). Each structure was assessed for structural modeling reliability using a *Z*-score approach of a knowledge-based force field YASARA2 (60) relative to all solved structures of the PDB. Models were generated for both human and mouse and the structures aligned using MUSTANG to calculate sequence and atom alignments [in root mean square deviation (RMSD)]. Each protein was also run for 10 ns of molecular dynamic simulations (MDS) using the AMBER03 force field (61) to assess the average movement in RMSD of the carbon alpha positions throughout the proteins. For all four proteins, evolution was mapped onto protein structures using the sequence alignments above with the ConSurf tools (62). Homology modeling for the *Drosophila* UPD2 and Dome proteins was performed using YASARA (60) and structure scores calculated with the YASARA2 knowledge-based force field. BLAST analysis was performed for invertebrate genomes using all available sequences of ENSEMBL Metazoa BLAST (http://metazoa.ensembl.org/Multi/Tools/Blast?db=core) including Arthopoda, Nematoda, Lophotrochozoa, and Cnidaria. Sequences for metazoa, fungi, and plant endospanin orthologs (*LEPROT* and *LEPROTL1* genes) were also pulled for ENSEMBL annotated orthologs.

**Table 1:** **Vertebrate *LEP, LEPR, LEPROT*, and *LEPROTL1* genes analyzed**.

*Gene*	Open reading frame sequences	AA start	AA end	Codons analyzed	Human model *Z*-score	Mouse–human homology (%)	Mouse–human alignment [root mean square deviation (RMSD), Å]	Molecular dynamic simulations carbon alpha (RMSD, Å)	Conserved posttranslational modifications
*LEP*	93	22	167	13,578	0.28	84.93	0.342	1.33	C117, C167
*LEPR*	89	29	1,158	100,570	−3	75.35	0.457	2.06	C196, N347, C352, C412, C413, C418, C447, C473, N624, N659, N688, N728, S882, Y986, Y1079, Y1141
*LEPROT*	150	1	131	19,650	−0.56	94.66	0.346	2.85	–

*Z-score is an indicator of how close (number of SDs from the mean) the predicted model fits chemical properties of all previously solved protein structures; RMSD is a measure of average distance between predicted models and native structures*.

**Figure 4:**
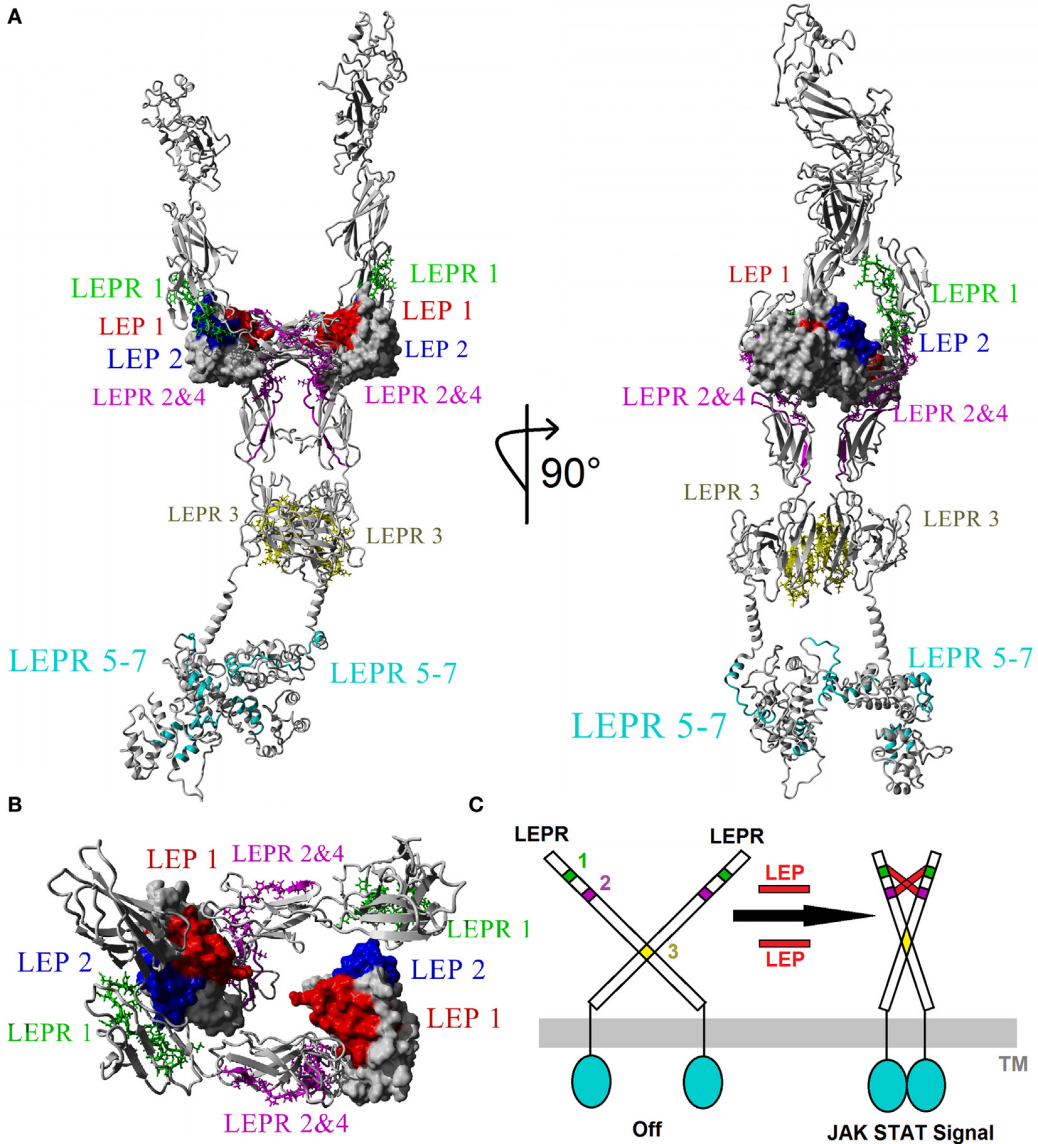
**Modeling the 2xLEP–2xLEPR interaction**. **(A)** Each of the top motifs for leptin (LEP) and leptin receptor (LEPR) are colored in respective color coding. LEP: motif 1, red; motif 2, blue. LEPR: motif 1, green; motif 2 and 4, magenta; motif 3, yellow; motif 5–7, cyan. **(B)** Magnified view of the Ig-like and LBD of LEPR showing the 2–2 interaction model based on vertebrate evolution. **(C)** Model of endogenous dimerized LEPR being activated by LEP binding.

We hypothesize that the physiological effects of leptin are induced *via* binding with leptin receptor in a 2–2 molecular interaction, resulting in conformational stability to already dimerized receptors (37, 44, 65–67). There is evidence of higher order oligomerization states such as that of 4:4 stoichiometry (66, 68); however, very little is known about the structural basis for these states. Merging conserved motifs into the model of leptin–leptin receptor interaction, a 2–2 molecular interaction model was created (Figure 4A) using previous structures as a guide (66). When viewing the entire leptin receptor protein (Figure 4A), docking of leptin to leptin receptor accounted for all motifs. Motif 1 of leptin (red, Figure 4A) interacts with motifs 2 and 4 (magenta, Figure 4A) of leptin receptor, while motif 2 of leptin (blue, Figure 4A) interacts with motif 1 of LEPR (green, Figure 4A). Motif 3 of leptin receptor (yellow, Figure 4A) falls in the fibronectin type III 3 domain, known to control non-LEP-dependent dimerization of LEPR (67, 69, 70). Our models suggest with high probability that this motif contributes to dimerization of the receptor. In this dimer model, LEPR exists on the surface of cells as a dimer controlled by the conserved motif 3, such that the intracellular regions are not in close proximity to each other (Figure 4B). On two leptin molecules binding, the receptor is hinged by motif 3 (yellow, Figure 4C) to bring together motifs 1, 2, and 4 of LEPR to LEP motifs 1 and 2, resulting in intracellular domains of LEPR brought into close proximity for JAK and STAT activation (Figure 4C).

## Evolution of Leptin Signaling: Invertebrate Leptin Signaling Genes

Rajan and Perrimon in 2012 described what they thought was a homologous leptin system in *Drosophila melanogaster* (71), through Unpaired 2 (*Upd2*) and Domeless (*Dome*) proteins. Similar to vertebrate *LEPR*s in fish (72), chicken (73), pig (74), cow (75), rat (76), and human (77), the Dome protein of *Drosophila* is critical for germline and follicle cell development through UPD signaling (78). Recent reports of a putative leptin signaling system in *D. melanogaster* through the UPD2 and Dome proteins (71), which are associated with phenotypes from tissue development (79), memory (80), and reproductive systems (78), proposes conserved leptin signaling components in invertebrates. Overlapping functions of vertebrate leptin receptor and Dome proteins suggest possible conserved tertiary structure.

**Figure 5:**
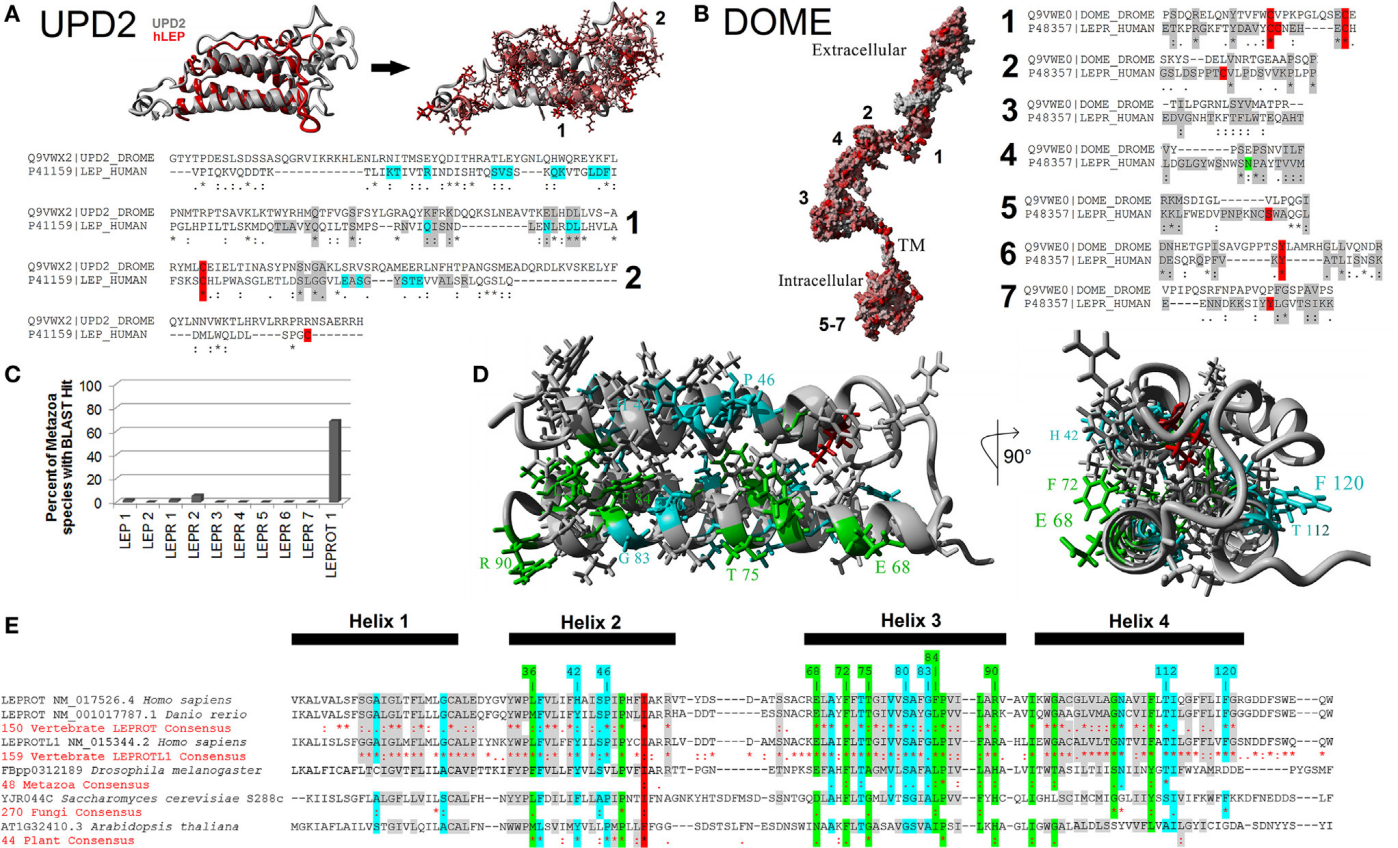
**Defining the metazoan leptin system**. **(A)** UPD2 model (gray) aligned with human LEP (hLEP, red). To the right of the structure overlay are identified amino acids in red that are conserved in the two proteins. Below the models are sequence alignments showing amino acids conserved in the top two motifs (gray), conserved posttranslational modifications (PTMs) (red), and known sites to interact with the receptor (cyan). **(B)** Dome model with amino acids in red conserved with human leptin receptor (LEPR). To the right of the model are sequence alignments showing amino acids conserved in the top seven motifs (gray) and conserved PTMs (red). **(C)** Using the top motifs of LEP, LEPR, and LEPROT, BLAST data for each in invertebrate genomes. **(D,E)** Models of LEPROT, now known as endospanin 1 **(D)**, with conserved amino acids identified **(E)**. Amino acids in red are conserved in all sequences, those in green conserved in at least four of the five groups, those in cyan conserved in at least three groups, and those in gray conserved in at least two groups.

To test homology between vertebrate and invertebrate systems, we modeled UPD2 (Figure 5A) and Dome proteins (Figure 5B) using our LEP:LEPR models and evaluated conservation of vertebrate motifs in the fly proteins. The UPD2 protein four-helix bundle was homologous to hLEP with some conserved amino acids contributing to packing and others that were surface exposed (Figure 5A). Structural alignments of hLEP to UPD2 had 8.24% homology and an alpha carbon average RMSD of 1.627 Å. The UPD2 model had a *z*-score of −1.375, which suggests that the model contains behaviors similar to most known protein structures. Aligning sequence of LEP to UPD2, 9 of 20 amino acids contributing to LEP–LEPR interaction were conserved in UPD2 (cyan, Figure 5A), fitting within our expectations based on zebrafish LEP modeling (37). Motif 1 of vertebrate LEP had 8 of 16 conserved amino acids, while motif 2 had 3 of 13 conserved amino acids. These data suggest a high probability of similar fold between leptin and UPD2 with a high number of amino acids conserved that are known to interact with leptin receptor, including motif 1 generated from our vertebrate evolutionary analysis.

The DOME and hLEPR models align with 22.94% homology and an average RMSD of alpha carbons of 0.823 Å (Figure 5B). To refine the functional conservation of DOME to hLEPR, we analyzed each of the top seven vertebrate motifs of LEPR. Each of the seven motifs of vertebrate *LEP* were found in the Dome sequence. Motif 1 had 6 of 14 critical amino acids conserved including two cysteine amino acids involved in disulfide bond formation. Motif 2 and 4 involved in the main interaction with LEP had hydrophobic and structural amino acids conserved with the vertebrate sequences. Motif 3 involved in non-LEP-dependent LEPR dimerization had three critical hydrophobic amino acids conserved. Of the intracellular three motifs 5–7, motif 6 was the most highly conserved including the known tyrosine phosphorylation site. To our knowledge, the combination of these seven motifs is not found in any other human protein, thus the high conservation of these motifs in Dome supports the assertion that this is indeed a homolog of vertebrate LEPR.

To probe the existence of the leptin signaling genes in other invertebrate genomes, a BLAST approach for the top motifs was used (Figure 5C). BLAST analysis of 54 invertebrate genomes was unable to identify invertebrate homologs. This is likely due to insertions and deletions seen in the motif alignments of Upd2 and Dome (Figures 5A,B), decreasing success of BLAST approaches. By using Ensembl Metazoa annotation tools (81), Upd2 homologs were only identified in the 12 sequenced *Drosophila* species, with no other invertebrates having annotated homologs. The Dome protein, however, has homologs found in 48 species of invertebrates according to ENSEMBL (http://metazoa.ensembl.org/Drosophila_melanogaster/Gene/Compara_Ortholog?db=core;g=FBgn0043903;r=X:19676061-19683518;t=FBtr0074756), with 22 being found as similar size of human LEPR and *D. melanogaster* Dome proteins. Outside of invertebrates, no homologs of *Upd2* or *Dome* are yet reported. Contrary to *LEP* and *LEPR*, the *LEPROT* gene is found in many species from invertebrates to plants to fungi (Figures 5D,E).

## Evolution of Leptin Signaling: Endospanin

Three years after the discovery of the leptin, Bailleul et al. established that the human *LEPR* transcribes a second, non-leptin receptor gene product (82). Initially named leptin receptor gene-related protein (*OB-RGRP*) or *LEPROT* (83), it is transcribed from an alternate AUG within the leptin receptor gene. The alternate start site is out of frame with the leptin receptor transcript, such that it produces a 131 amino acid protein that shares no primary sequence with *LEPR*.

*LEPROT* [recently renamed endospanin (84)] and its paralog *LEPROTL1* (endospanin 2) are homologous with the yeast vesicle trafficking gene *VPS55* (85). Knockout or disruption of VPS55 in yeast results in generally altered endosomal/vacuole trafficking (85, 86). In vertebrates, endospanin is proposed to specifically regulate endosomal trafficking and surface expression of the leptin receptor. Knockout LEPROT mice express more leptin receptors on the cell surface than wild-type, which makes them hyperresponsive to leptin and resistant to diet-induced obesity (87–89). Further, LEPR protein expression and *LEPROT* genomic copy number are negatively correlated in humans (90), and *LEPROT* may control tissue-specific expression of *LEPR* (91). Both endospanins 1 and 2 are known to interact with Rab13 and Rab8 (92), small G-proteins critical for trafficking between the trans-Golgi network and other cell compartments (93, 94). This suggests that endospanin1/2’s role is larger than just regulation of leptin receptor protein.

Is endospanin function conserved among vertebrates? While the BLAST approach did not identify invertebrate *LEP* and *LEPR*, the Ensembl Metazoa annotation (81) identified 48 invertebrate genomes as containing *LEPROT* homologous proteins. Further, 270 sequenced fungi and 44 sequenced plants contain a *LEPROT* homolog. We combined all of these sequences with 150 and 159 vertebrate LEPROT and LEPROTL1 sequences both to build the first tertiary structure prediction for endospanin and to determine critically conserved amino acids throughout eukaryote evolution (Figures 3 and 5D,E).

One amino acid is conserved in all 671 sequences studied (red), 13 amino acids are conserved in at least 4 of the 5 taxa (green), 16 in at least 3 of the 5 organism groupings (cyan), and 45 conserved in at least 2 of the 5 organism groupings (gray, Figures 5D,E). Using the total of 140 positions in the sequence alignment as shown, red represents 0.7%, green represents 9.3%, cyan represents 11.4%, and gray represents 32.1%, and thus 53.5% of the protein is identified to maintain conservation in at least one of the groupings. This value far exceeds that of LEPR and Dome proteins. Endospanin 1 protein contains a four-helix transmembrane bundle with high conservation of a hydrophobic core of the protein (Figure 5D). Noting conserved amino acids on our four-helix model (Figure 5D), 12 amino acids were conserved and surface exposed at positions 36, 42, 46, 68, 72, 75, 80, 83, 84, 90, 112, and 120 using the human LEPROT numbering (Figure 5E). These residues make up one side of the helix, suggesting possible interaction with another protein at this site.

**Figure 6:**
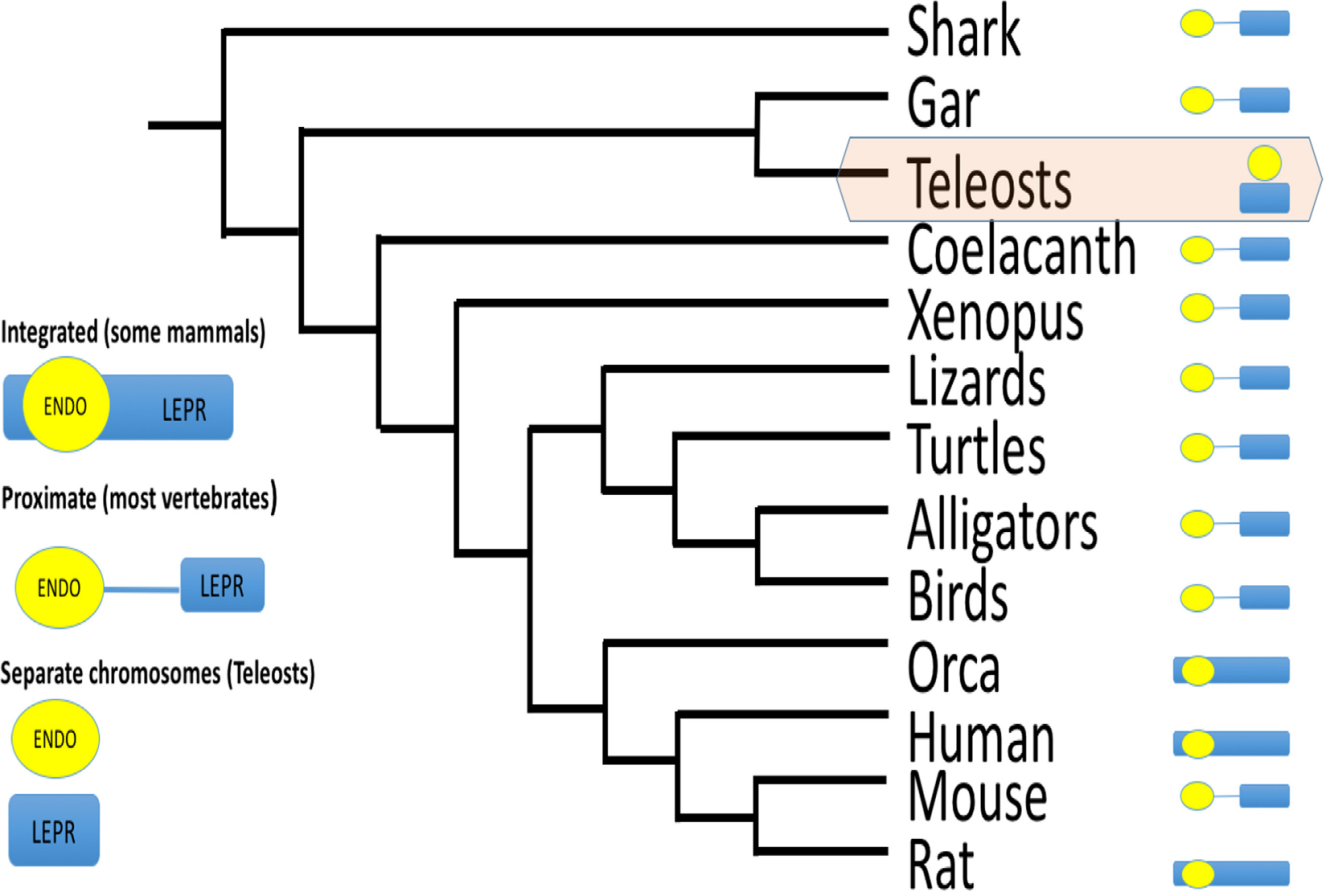
**Schematic of gene order for endospanin and leptin receptor (LEPR) among vertebrates**. For most vertebrate classes, endospanin (*LEPROT*) is either embedded within the *LEPR* gene, or within 150,000 bp, and without any gene between *LEPROT* and *LEPR*. For teleost fishes only, *LEPROT* and *LEPR* are on separate chromosomes. Data mined from Genbank queries. For example, LEPR search term returns chromosome 1, acc# NC_000001.11 for human *LEPR*, which also maps *LEPROT* within the human *LEPR* sequence, and chromosome 6, acc# NC_07117.6 for zebrafish *LEPR* but chromosome 2, acc# NC_007113.6 for zebrafish *LEPROT*.

Another aspect of *LEPROT* genomics likely affects its influence on *LEPR* functional expression (i.e., on the surface of the cell). LEPROT’s original designation was as the “leptin receptor overlapping transcript” (82), indicating that *LEPROT* overlapped *LEPR*. Surveying Genbank for *LEPROT* and *LEPR* loci in all vertebrate classes, *LEPROT* overlaps or is adjacent to *LEPR* (within 150,000 bp and no intervening gene) in all cases. The one exception is teleost fishes, where *LEPROT* and *LEPR* are on different chromosomes (Figure 6). Gene proximity affects transcription rates (95). Given the high conservation of endospanin sequence, the conservation of its synteny with leptin receptor, and its effect on leptin receptor functional expression (87–89), it is likely that *LEPROT* and *LEPR* coevolved. We assert that because that synteny is broken in teleosts, it may be that control of leptin receptor expression is unique for teleosts among vertebrates.

## Evolution of Leptin Signaling: What Does Gene Evolution Tell Us about Human Leptin Signaling?

Uncovering the evolutionary history of leptin signaling genes and modeling their structure is valuable as a self-contained enterprise, because it sets the stage for understanding functional differences among taxa. However, knowing how these genes are represented among vertebrates also has translational value. Modeling of *Drosophila* Dome as a leptin receptor and finding Dome homologs among other invertebrates provide an avenue for studying leptin signaling in other model systems. How changes in leptin signaling contribute to obesity is certainly complex, with interacting endocrine, neurological, epigenetic, and environmental variables. Added to this complexity is the interaction of multiple leptin receptor isoforms in transporting leptin across the blood–brain barrier. Decreased leptin signaling in the presence of high titers of circulating leptin, or leptin insensitivity/resistance, is often implicated as contributing to human obesity (96, 97). There is growing consensus that reduced blood–brain transport of leptin is a contributing factor to leptin insensitivity in the face of high leptin titers caused by obesity [reviewed in Ref. (98)]. Transport of leptin across the blood–brain barrier is facilitated by leptin receptors with short intracellular domains [commonly referred to as the ObRa paralog, as opposed to the ObRb paralog, which has a complete intracellular domain and is capable of mediating intracellular signaling (99, 100)]. This transport can be inhibited by a soluble form of the leptin receptor (ObRe), capable of binding leptin in serum (101, reviewed in 102). Sequencing cDNAs indicates that these isoforms are the result of alternate splicing of *LEPR* [e.g., Ref. (103)]; however, soluble receptors can also result from cleaving of membrane bound leptin receptors (102). All studies on leptin resistance (to our knowledge) are conducted in mammal models. Comparative study across model systems has the potential to illuminate how receptor paralog diversity contributes to leptin sensitivity. Given that endospanin controls surface expression of long-form LEPR [ObRb (82, 89)], it may also control expression of the other leptin receptor paralogs. Because endospanin is highly conserved, it represents an opportunity to study leptin sensitivity across models and an avenue to explore for human obesity treatment.

## Leptin as an Adipostat: Lack of Evidence in Fishes

Recently, we made an argument that leptin in fishes does not fit the adipostat model proposed for mammals (16). Importantly, we are distinguishing between leptin’s proposed *adipostat* function and its *anorexigen* function. Leptin’s anorexigenic function is well documented among fishes (104, 105), amphibians (3), birds (although with non-homologous leptin) (106), lizards (25), and mammals (4). However, central to the adipostat model is the idea that serum leptin is proportional to total adipose stores, because adipose is the major producer of leptin in mammals (4–6). Kurokawa’s seminal study first noted that the primary tissue expressing leptin in fish was liver and not adipose (2). This was confirmed in many [e.g., Ref. (31–33)] but not all (107) fish species. Instead of decreasing as fat stores are depleted (as predicted by adipostat), plasma leptin consistently increases with fasting in salmonids (108–110) and flounder (111). Striberny et al. (112) found no evidence that this change in circulating leptin titer was mediated by the CNS. Further, Arctic charr will spontaneously stop feeding in winter even while leptin titers are falling and even if presented with food (11), but will resume eating during the time of year when leptin concentrations are rising (113, 114). The observation that leptin increases at the end of a long fast in fishes runs counter to leptin’s documented anorexigenic effects (above). It may be that plasma leptin titers in fasting fish are below the threshold that triggers an anorexigenic response. It is also possible that leptin injections result in supraphysiological concentrations of the hormone in serum, eliciting a response not seen with “normal” leptin signaling (115) and eliciting responses even with artifactual leptins (116). In fishes, increasing serum leptin commonly is interpreted as a signal to mobilize lipid stores in preparation for reproduction, rather than a response to fasting *per se* (113, 114).

Clearly, a decreasing leptin signal during winter and increase prior to reproduction is not consistent with the adipostat model proposed for mammals (2–4). The majority of leptin studies are done on rodents (16), and as such our view of leptin as an adipostat is likely biased by those studies. Rodents have high mass-specific metabolic rates and can only fast for hours, whereas hibernating mammals and ectothermic fishes routinely fast for months. Although leptin is thought to drive the prehibernation anorexia of some, but not all hibernating mammals [reviewed in Ref. (117)], organisms with life histories that are distinctly seasonal (but not necessarily hibernating) may change their set point for leptin sensitivity to accommodate different levels of activity and food availability between seasons (97, 112, 118, 119); thus, an adipostat as described for rodents may not be adaptive for fishes.

Total lipid stores (summing all tissues) clearly are not reflected in serum leptin (as evidenced by fasting fish that increase leptin titers above). However, many researchers (including us) have assumed that liver or gonad is the tissue that contributes to the bulk of serum leptin (because it gives the highest qPCR signal), but that may not be true. Salmonid adipocytes express detectable leptin (110, 120–123), and adipocytes cultured from food-restricted fish secrete significantly more leptin than those from fed fish, reflecting the response of the whole organism (110). Recent knockout models either affect adipose tissue [medaka *lepr* knockout (124)] or do not [zebrafish *lep* knockout (125)]. It may be that the bulk of leptin’s serum titer results from expression from liver, but that other tissues express leptin for autocrine/paracrine roles, as proposed for birds (see below). Well-controlled immunological studies, such as those for salmonids and tilapia (110, 126), are needed for a diversity of teleosts, along with how each tissue contributes to the serum/local pool under various physiological conditions.

How does the status of the adipostat model in fishes affect leptin as an adipostat in other vertebrates? We now know that human obesity is influenced by changes in food perception and metabolism after weight loss (127), and therefore a simplistic adipostat feedback loop does not adequately model human phenotypes. Documenting the response of appetite and leptin across vertebrates argues that it is possible to adjust leptin sensitivity, and even presents possible mechanisms for how sensitivity changes (e.g. endospanin).

## Emerging Non-Mammal Models of Leptin Signaling

The obese (*ob/ob)* mouse, a long-standing model of human obesity (128), gained favor for leptin studies after Freidman’s laboratory cloned the truncated *LEP* gene (1). Together with the diabetic (*db/db*) mouse, a *LEPR*-deficient model, leptin administration effects have been demonstrated repeatedly. Intraperitoneal leptin injections in *ob/ob* mice causes 30% decrease in body mass, and *db/db* mice are similar to controls (5). Leptin’s pleiotropy was detailed using these models, and as a result, we now know that leptin affects reproduction, immune function, bone growth/resorption, and metabolic rate [reviewed in Ref. (129–132)]. The long-term normalizing effects of peripheral leptin injections on hLEP congenital deficiency reflect those in the *ob/ob* mouse [reviewed in Ref. (133)]. There are lines of fish (134) and birds (135) selected for high and low adiposity; however, few *LEP* and *LEPR*-null models are available for comparative (leptin) studies.

Our group used morpholino knockdown to generate zebrafish embryos with reduced leptin signaling (136, 137). We documented severe developmental defects in response to knockdown of *LEPA* or *LEPR*. Morphants were characterized by malformed sensory structures, bent notochord, poor yolk absorption, and low metabolic rate; these effects were rescued by coinjection of recombinant zebrafish leptin (136, 137). Microarray analysis of leptin-A “morphant” and “rescue” expression data identified differentially expressed genes that correspond to leptin signal transduction pathways [GnRH signaling, fatty acid metabolism, glycolysis/gluconeogenesis, MAP kinase, phosphoinositol signaling (138)]. The recent availability of CRISPR technology allowed direct comparison of zebrafish gene knockdown vs. gene knockout. “Morphant” and “mutant” phenotypes generally do not agree when targeting the same gene; typically morphants do not emulate mutant phenotypes (139, 140). Zebrafish morphants targeting (apparently) unrelated genes often share combinations of morphological markers ranging from disrupted eye, ear, and brain development; irregular body/tail curvature; or enlarged yolk (139, 141, 142). Non-specific MO off-target activity upregulates zebrafish *tp53*, which may induce apoptosis and global changes in gene expression (139, 143). For these reasons, we are hesitant to pursue antisense technologies. Similar to recent work by other laboratories, we are opting for knockout technologies as a means to generate comparative null models for many leptin signaling genes. Chisada et al. produced the first *LEPR* mutant fish, using the TILLING approach in medaka (124). Adult medaka *LEPR* mutants are hyperphagic, have elevated NPYa and AGRP, and decreased POMC mRNAs. Liver and muscle lipid does not increase in the *LEPR* mutants, but they accumulate visceral fat as adults (124). The medaka data are consistent with a mammalian adipostat model, but zebrafish are not. Michel et al. recently characterized an established (144) *LEPR* TILLING mutant in zebrafish and also generated CRISPR mutants for *LEPA, LEPB*, and *LEPR* (125). *LEPR*-null adults have no differences from wild-type in adiposity, body size, growth rate, mating success, or feeding behavior. However, *LEPR* mutants have altered glucose metabolism, and both *LEPR* and *LEPA* larvae have increased β-cell number (125).

## Conclusion and Future Directions

Comparative leptin endocrinology has matured in the 11 years since the first non-mammal leptin was cloned. All major vertebrate classes are now represented in cloned leptins and leptin receptors, and investigation of invertebrate leptin signaling is beginning. Protein structures have been modeled for leptin, leptin receptor, and endospanins across an extensive evolutionary timescale, but models (although useful) are simply predictions to be tested. Now that we have identified conserved motifs and conserved sites of leptin–leptin receptor interaction, these predictions should be tested with *in vitro* functional assays.

The bird leptin problem has been solved in a genomic sense, but is just initiating physiologically. Now that the bird receptor assays (49) can be used with homologous ligands (hopefully soon), we should learn if birds are truly different among vertebrates in leptin signaling. We assert that understanding the endocrinology of how birds manipulate lipid stores will pay dividends in comparative endocrinology, agriculture, and human disease. Robust data from decades of research demonstrate that many species of birds perform large-scale manipulation of energy stores, and preliminary (but compelling) data indicate that they are doing so either without leptin (or other known adipokines) or by using leptin in a fundamentally different way (e.g., through an uncharacterized pathway). If leptin signaling in birds is truly different, it means that there is another way that vertebrates manipulate energy stores and thus potentially new avenues to pursue that will help us understand human obesity.

Amphibian leptin models are well developed with homologous recombinant leptin and receptor assays (15, 24), but relatively little is known for reptiles. These groups appear to adhere to the lipostat model, while birds and teleost fishes may not. As such, more species diversity in amphibian and reptile leptin studies could be very important in understanding leptin function as an adipostat.

In fishes, we are now past the point where one’s fish species of choice can be interpreted as representative of all fishes. Given that bony fishes have been on the planet ~370 MY longer than modern mammals (www.timetree.org), it is not surprising that they may be diverse in their leptin signaling. Phylogenetic analyses make it clear that teleost fishes are diverse in the structures of their leptin, leptin receptor, and *LEPROT* genes, and it is likely that reported differences among species represent true species divergence rather than methodological idiosyncrasies. Although we argue that there is a lack of evidence for adipostat function in fishes, the future may reveal that an “origin(s)” of that status within a fish clade, and we simply need to sample fish diversity more completely (e.g., non-teleost fishes need attention).

If we are to move forward, we must have comparable variables to assess species diversity. As such, reliance on relative qPCR for expression data does not allow quantitative comparisons among species; the community needs well-validated ELISAs (such as that developed for salmonids and tilapia) for multiple species. In this same light, the non-coding regions near leptin and leptin receptor need to be studied with more detail to gain an understanding of how expression is controlled throughout evolution. We need to pursue knockout models in non-mammals for laboratory approaches comparable to those using *ob/ob* and *db/db* mice. Finally, we need to measure leptin signaling responses of unmanipulated animals in the field and take advantage of the tremendous diversity of life histories that are well suited for leptin questions [The Krogh Principle (145)]. In doing so, the comparative community will contribute to understanding of human obesity similar to how *Drosophila* studies contributed to genetics, how shark-rectal gland contributed to kidney function, or how the squid giant axon contributed to neurobiology.

## Ethics Statement

All procedures performed by the authors that used animals were approved by the institution’s IACUC.

## Author Contributions

All authors contributed to drafting and editing of the manuscript. JWP completed all molecular modeling.

## Conflict of Interest Statement

The authors declare that the research was conducted in the absence of any commercial or financial relationships that could be construed as a potential conflict of interest. The reviewer RD and handling editor declared their shared affiliation, and the handling editor states that the process nevertheless met the standards of a fair and objective review.
